# Transoral Outlet Reduction for Dumping Syndrome After Roux-En-Y Gastric Bypass: a Comprehensive Systematic Review and Meta-Analysis

**DOI:** 10.1007/s11695-025-08275-9

**Published:** 2025-09-27

**Authors:** Azizullah Beran, Daryl Ramai, Almaza Albakri, Khaled Alchirazi, Nasir Saleem, Mark A. Gromski

**Affiliations:** 1 Division of Gastroenterology and Hepatology, Indiana University School of Medicine, Indianapolis, IN USA; 2 Gastroenterology, Brigham and Women’s Hospital, Boston, MA USA; 3 Internal Medicine, Cleveland Clinic, Cleveland, OH USA; 4 Gastroenterology, Aurora St. Luke’s Medical Center, Milwaukee, MI USA

**Keywords:** Transoral outlet reduction, Gastrojejunal anastomosis revision, Roux-en-Y gastric bypass, Dumping syndrome

## Abstract

**Introduction:**

Dumping syndrome is a complication of Roux-en-Y gastric bypass (RYGB) surgery that can significantly affect quality of life. Transoral outlet reduction (TORe) is a minimally invasive endoscopic procedure that reduces the size of the gastrojejunal anastomosis (GJA) through ablation and/or endoscopic suturing, primarily used to address weight regain after RYGB. Emerging evidence highlights the feasibility and safety of TORe as a treatment for medically refractory dumping syndrome. This meta-analysis aims to evaluate the efficacy and safety of TORe for dumping syndrome.

**Methods:**

A systematic search of PubMed, Embase, and Web of Science was conducted through December 2024. Primary outcomes included clinical success (defined as sustained symptom improvement without requiring repeat TORe or revisional surgery at last follow-up) and the mean difference in pre- and post-procedural Sigstad’s score assessments. Secondary outcomes included rates of post-TORe surgery, repeat TORe, and serious adverse events. Pooled rate estimates and mean differences (MD) with the corresponding 95% confidence intervals (CI) were calculated using random-effects models.

**Results:**

Six studies with 333 post-RYGB patients with dumping syndrome were included. The pooled clinical success of TORe was 83% (95% CI 71%-90%, I^2^ = 74%). Furthermore, TORe resulted in a significant improvement in Sigstad’s score (MD − 11.12 [95% CI -15.33 to − 6.91], *P* < 0.001, I^2^ = 89%). The rate of serious adverse events was 3% (95% CI 0.7%-12.4%, I^2^ = 68%).

**Conclusions:**

Our findings suggest that TORe is a safe and effective minimally invasive treatment for patients with medically refractory dumping syndrome. Further prospective studies with longer follow-up durations are warranted to validate these findings.

**Supplementary Information:**

The online version contains supplementary material available at 10.1007/s11695-025-08275-9.

## Introduction

Obesity is a chronic, relapsing disease that has reached pandemic levels worldwide [1]. In response, a growing number of individuals are turning to bariatric surgery, which is the most effective long-term intervention for sustained weight loss and improvement in obesity-related comorbidities [2]. Roux-en-Y gastric bypass (RYGB) remains one of the most widely performed and well-established bariatric surgeries for weight loss [3]. However, despite its durable efficacy, RYGB is associated with adverse events such as marginal ulcer, weight regain and dumping syndrome [4, 5].

Dumping syndrome, a frequent but underdiagnosed complication of RYGB, affects up to 25–50% of patients and can significantly reduce health-related quality of life [6–8]. This condition is characterized by recurrent episodes of postprandial hypoglycemia, presenting with a constellation of gastrointestinal symptoms (abdominal pain, bloating, borborygmi, nausea and diarrhea) and vasomotor symptoms (flushing, palpitations, perspiration, tachycardia, hypotension, fatigue, rarely, syncope) [9]. Dumping syndrome is classified into two types: early dumping syndrome, which typically occurs within one hour after eating and is believed to result from rapid fluid shifts, and late dumping syndrome, which manifests 1–3 h post-meal and is thought to stem from an incretin-driven hyperinsulinemia after carbohydrate ingestion [9]. The Sigstad scoring system is typically used to assess the severity of dumping syndrome (Table 1) [10].

**Table 1:** Sigstad scoring system for dumping syndrome

Postprandial symptoms	Score
Shock	+ 5
Faintness, syncope, or unconsciousness	+ 4
Desire to lie down	+ 4
Dyspnea	+ 3
Weakness	+ 3
Sleepiness, apathy	+ 3
Palpitations	+ 3
Restlessness	+ 2
Dizziness	+ 2
Headaches	+ 1
Warm, clammy skin, or pallor	+ 1
Nausea	+ 1
Abdominal fullness	+ 1
Borborygmi	+ 1
Belching	−1
Vomiting	−4

Dietary modifications are the mainstay treatment for dumping syndrome, which includes small frequent meals and avoiding simple carbohydrates [9]. Medications such as acarbose can be used as second line when dietary modifications are insufficient [9]. In refractory cases, surgical revisional procedures may be considered; however, these can be technically difficult in the presence of altered anatomy and adhesions and are associated with an increased risk of complications [9, 11]. Transoral outlet reduction (TORe) is a minimally invasive endoscopic procedure that reduces the size of the post-RYGB gastrojejunal anastomosis (GJA) through ablation and/or endoscopic suturing, primarily used to address weight regain after RYGB [12]. Emerging evidence highlights the feasibility and safety of TORe as a treatment for dumping syndrome that is refractory to dietary modifications and medical therapy [13]. This systematic review and meta-analysis aims to evaluate the efficacy and safety of TORe for managing dumping syndrome.

## Methods

### Data Sources and Search Strategy

We conducted a comprehensive search of PubMed, Embase, and Web of Science databases from inception through December 31 st, 2024 for published studies that assess the efficacy and safety of TORe for management of dumping syndrome. We also manually identified additional relevant studies using the references of included studies. The following MeSH terms were used: (“transoral outlet reduction”) and (“dumping syndrome”). Supplementary Table 1 describes the full search terms used in each database searched. We followed the Preferred Reporting Items for Systematic Reviews and Meta-Analyses (PRISMA) guidelines to select the final studies [14]. No Institutional Review Board or Ethics Committee approval required for this study. Two reviewers (AB and AA) independently screened and selected the potentially included studies. Discrepancies were addressed by a third reviewer (DR).

### Eligibility Criteria

All peer-reviewed studies (observational studies or randomized controlled trials) that assessed the efficacy and safety outcomes of TORe procedure for managing dumping syndrome following RYGB were eligible for inclusion. Case reports, case series, and conference abstracts were excluded.

### Data Extraction

Data on study and patient characteristics and outcome measures were extracted by two independent reviewers (AB and AA). Extracted study characteristics included country of origin, study period and design, sample size, female gender percentage, age, baseline body mass index (BMI), GJA size (pre- and post-TORe), device type, procedure duration, number of sutures, pattern of suturing, follow-up period, proportion of weekend admissions, and reported outcomes (clinical success, pre- and post-TORe Sigstad’s score, need for surgical intervention, need for repeat TORe, and serious adverse events).

### Outcomes and Definitions

The primary outcomes were clinical success and the mean difference in pre- and post-procedural Sigstad’s score assessments. Clinical success was defined as sustained symptom improvement without the need for repeat TORe or revisional surgery at the final follow-up. Secondary outcomes included rates of surgery and repeat TORe to address refractory symptoms, and post-procedural serious adverse events.

### Statistical Analysis

Pooled rate estimates and mean differences (MD) with the corresponding 95% confidence intervals (CI) were calculated using random-effects models. *P*-values < 0.05 were considered statistically significant. Statistical heterogeneity was evaluated using the I^2^ statistics and I^2^ value of ≥ 50% was considered significant heterogeneity. All statistical analyses were conducted via Comprehensive Meta-Analysis version 4. To further validate the robustness of our results, we conducted a leave-one-out sensitivity analysis for clinical success.

### Bias Assessment

Risk of bias was assessed using the Newcastle–Ottawa Assessment Scale for included studies [15]. Two authors (AB and DR) independently assessed each study for bias. Discrepancies were resolved by consensus. Publication bias was assessed qualitatively by visually assessing the funnel plot and quantitively using Egger’s regression analysis for clinical success.

## Results

### Study Selection

**Fig. 1:**
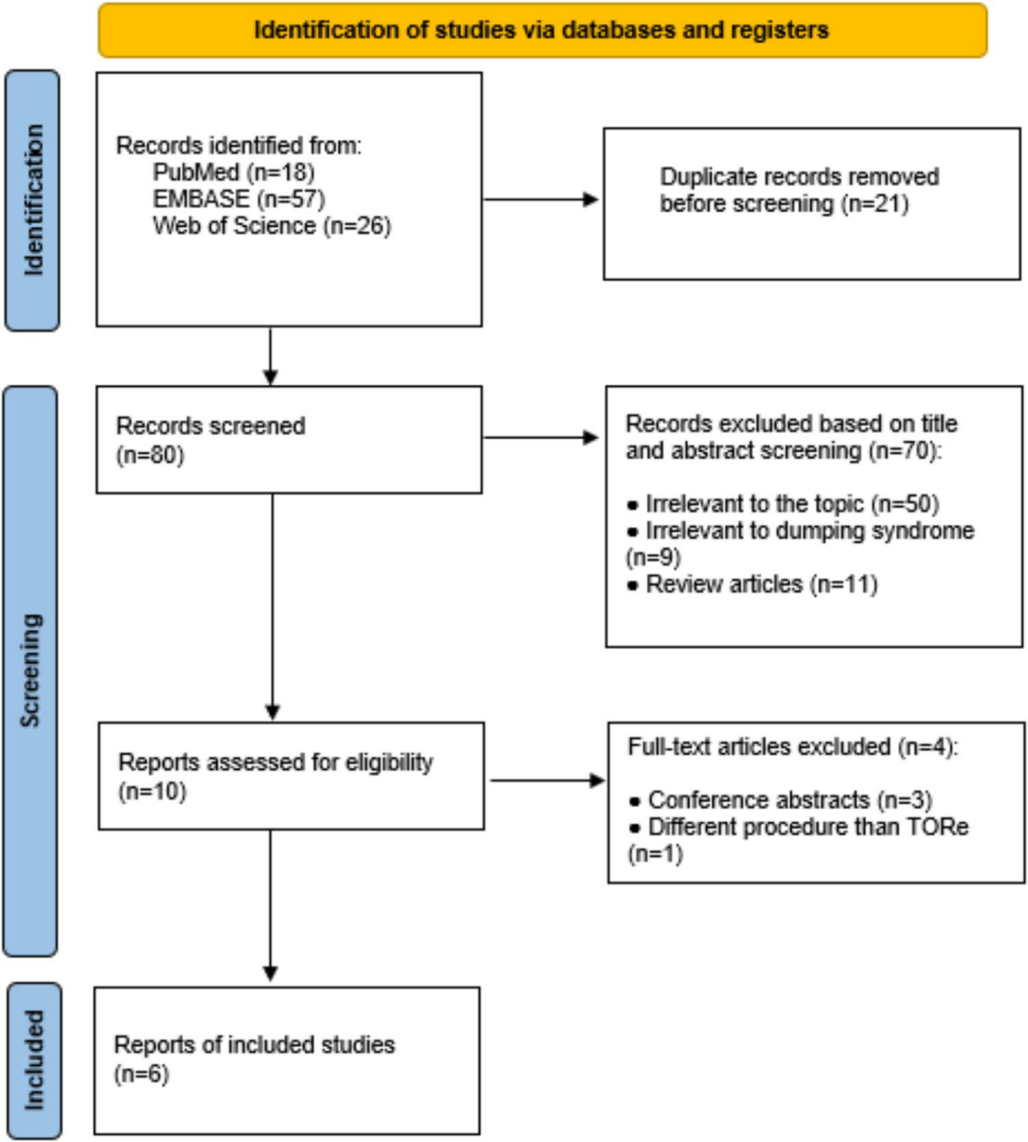
PRISMA flow diagram for the selection of studies

Of the 101 studies initially screened, 10 met the inclusion criteria for this systematic review. However, four of these were excluded—three were conference abstracts, and one evaluated outcomes of a procedure other than TORe. Consequently, six [13, 16–20] were included in the final meta-analysis. A visual summary of the selection process is provided in Fig. 1.

### Study and Patient Characteristics

Study and patient characteristics are summarized in Table 2. All studies were published between 2020 and 2024. Geographically, two [13, 18] studies were conducted in Switzerland, one [17] in Israel, one [16] in Italy, one [20] in United States, and one [19] was a multinational study involving the United States and Germany. In terms of study design, five [13, 16–18, 20] studies were retrospective cohort studies while one [19] was a prospective study. A total of 333 post-RYGB patients with dumping syndrome who underwent TORe were included in the six included studies [13, 16–20].

**Table 2:** Baseline characteristics and outcomes of the studies included in the meta-analysis

	Lovis, 2024	Petchers, 2022	Pontecorvi, 2023	Relly, 2021	Tsai, 2020	Vargas, 2020
Country of origin	Switzerland	United States	Italy	Israel	Switzerland	United States and Germany
Study design	Retrospective cohort	Retrospective cohort	Retrospective cohort	Retrospective cohort	Retrospective cohort	Prospective cohort
Study period	January 2015—December 2020	January 2013—December 2018	January 2015—June 2021	August 2018—September 2019	January 2016—August 2018	2014—2018
Total number of patients	9	98	58	13	40	115
Female gender, n (%)	8 (88.9%)	96 (98%)	NR	10 (76.9%)	27 (67.5%)	97 (84.3%)
Age, years, mean (range) or mean ± SD	46.0 (IQR 39.5)*	51 ± 9.9	NR	45.1 (25–56)	47.1 (22–75.4)	44.9 ± 9.2
Baseline BMI (kg/m2) or weight (kg), mean (range) or mean ± SD	34.5 (IQR 30.6–38.2) kg/m2*	36.15 ± 7.1 kg/m2	NR	33.5 (28.1–40.3) kg/m2	44.5 (35–60) kg/m2	98.4 ± 22.7 kg
Pre-TORe GJA size, mm, mean (range) or mean ± SD	30 (IQR 25–30)*	NR	NR	25.2 (15–30)	22.6 (18–35)	39.8 ± 6.7
Post-TORe GJA size, mm, mean (range)	9.5 (IQR 9.5–10)*	NR	NR	5.6 (5–10)	6.2 (4–13)	8–10
Device type	OverStitch (Apollo)	OverStitch (Apollo)	OverStitch (Apollo)	OverStitch (Apollo)	OverStitch (Apollo)	OverStitch (Apollo)
Procedure duration, minutes, mean (range)	30 (IQR 25–43)*	73 ± 27 (n = 59)	NR	47 (29–66)	18.5 (12–41)	38.9 ± 17.3
Number of sutures	3 (IQR 2–3)*	NR	2–3 sutures per patient	2 sutures	1* (range 1–3)	Mean 3 (range 2–5)
Pattern of suturing	Simple interrupted	Figure of 8	Simple interrupted	Figure of 8	NR	Simple interrupted or figure of 8
Follow-up period	12 months	3.45 years ± 1.7	6 months	6 months	Mean 12.5 months (range 1–33.8)	3 months
Clinical success, n (%)	7 (77.8%)	65/77 (84%)	40 (69%)	11 (84.6%)	30 (75%)	109 (94.8%)
Pre-TORe Sigstad’s score, mean ± SD	NR	NR	15 (11–18.5)*	19.4 ± 3.6	13.9 (range 0–28) (n = 25)	17.02 ± 6.1
Post-TORe Sigstad’s score, mean ± SD	NR	NR	3 (1–9.5)*	5.2 ± 5.5	8.6 (range 0–28) (n = 25)	2.55 ± 1.87
Surgical intervention, n (%)	2 (22.2%)	NR	NR	None	2 (5%)	3 (2.6%)
Repeat TORe, n (%)	None	NR	None	2 (15.4%)	9 (22.5%)	3 (2.6%)
Serious adverse events, n (%)	1 (11%) (stenosis)	1 (1%) (bleeding)	1 (1.7%) (abscess)	3 (23.1%) (nausea and vomiting requiring overnight hospitalization)	None	None

*Median

*BMI* body mass index, *GJA* gastrojejunal anastomosis, *n* sample size, *NR* not reported, *SD* standard deviation, *TORe* transoral outlet reduction

Among the five [16–20] studies that reported demographic and procedural details (*n* = 275 patients), 87% of patients were female, with a mean age of 47.5 ± 11.2 years and a mean BMI of 42.2 ± 10.3 kg/m^2^. Among the four [13, 17–19] studies reporting these data, the baseline mean diameter of the GJA was 34.2 ± 9.8 mm, and the mean baseline Sigstad score was 16.2 ± 7.4. All TORe procedures utilized the OverStitch (Apollo) device for endoscopic suturing. The mean procedure duration, reported across five [13, 17–20] studies, was 45.3 ± 27.4 min. In the small subset of patients with available data (*n* = 62), the mean post-procedural GJA diameter was reduced to 6.6 ± 4.01 mm. Follow-up durations across the studies ranged from 3 months to 3.4 years.

### Primary Outcomes: Clinical Success and Improvement in Sigstad’S Score

**Fig. 2:**
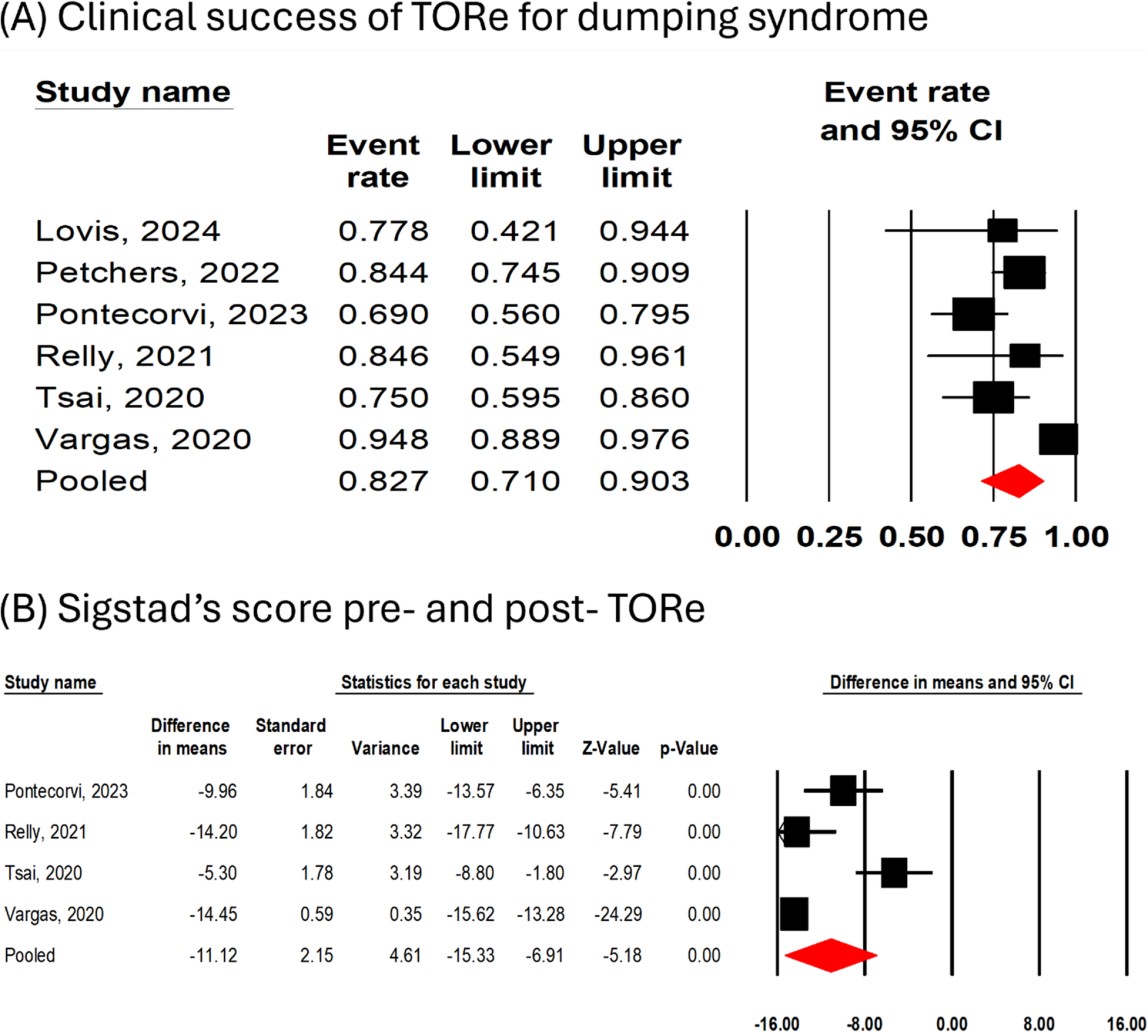
Forest plots for (**A**) clinical success of TORe for managing dumping syndrome and (**B**) pre- and post- TORe Sigstad score improvement

All [13, 16–20] studies (*n* = 312 patients, 21 patients lost to follow up) reported clinical success. The overall pooled clinical success was 83% (95% CI 71%−90%, I^2^ = 74%, Fig. 2A). A Leave-one-out sensitivity analysis for clinical success showed consistent results (Supplementary Fig. 1). Four [16–19] studies (*n* = 211 patients) reported pre-TORe and post-TORe Sigstad’s score. TORe resulted in a significant improvement in Sigstad’s score (MD − 11.12 [95% CI −15.33 to − 6.91], *P* < 0.001, I^2^ = 89%, Fig. 2B).

#### Secondary Outcomes: Rates of Surgery and Repeat Tore, and Serious Adverse Events

**Fig. 3:**
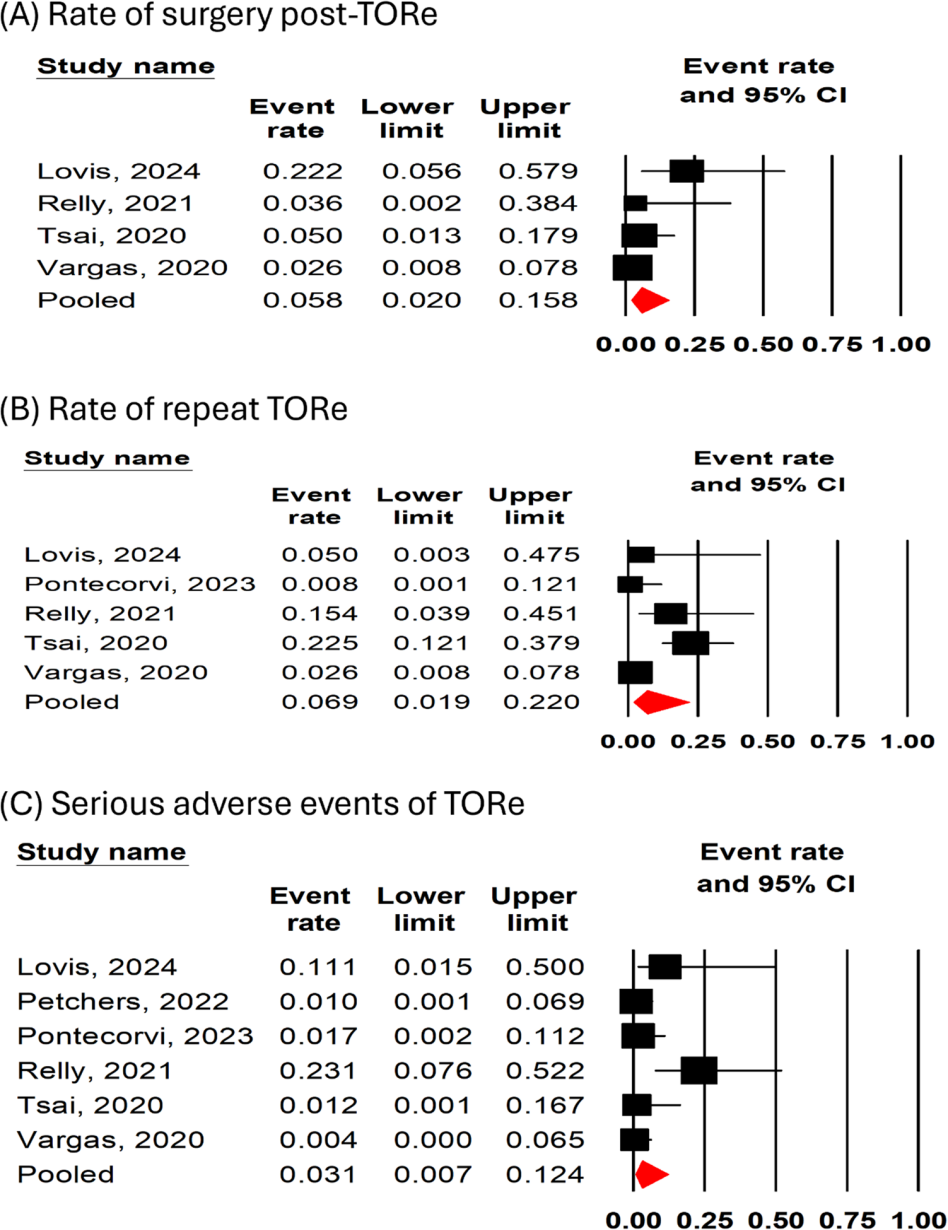
Forest plots for (**A**) surgery post-TORe, (**B**) repeat TORe, and (**C**) serious adverse events

Four [13, 17–19] studies (*n* = 177 patients) reported the rate of surgery post-TORe. The pooled rate of surgery post-TORe was 5.8% (95% CI 25–15.8%, I^2^ = 48%, Fig. 3A). All [13, 16–19] studies (*n* = 235 patients) reported the rate of repeat TORe. The pooled rate of repeat TORe was 6.9% (95% CI 1.9%−22%, I^2^ = 75%, Fig. 3B).). A Leave-one-out sensitivity analysis for repeat TORe showed consistent results (Supplementary Fig. 2). All [13, 16–20] studies (*n* = 333) reported the rate of serious adverse events. The pooled overall rate of serious adverse events was 3% (95% CI 0.7%−12.4%, I^2^ = 68%, Fig. 3C). Table 2 shows the details of serious adverse events reported in each study.

### Bias Assessment

The risk of bias was assessed and summarized in Supplementary Table 2. Of the six included studies, four [11, 12, 19, 21] were of low risk of bias while two [13, 20] was of high risk of bias in the assessment of outcomes. We found no evidence of publication bias for clinical success (*p* = 0.59) (Supplementary Fig. 3).

## Discussion

This systematic review and meta-analysis demonstrates that transoral outlet reduction (TORe) is a safe and effective minimally invasive therapeutic option for managing dumping syndrome. The pooled clinical success rate of TORe in treating refractory dumping syndrome was 83%, while the overall rate of serious adverse events remained low at 3%. These findings support the use of TORe as a valid intervention for patients with dumping syndrome who do not respond to lifestyle modifications and medical therapy, offering a less invasive alternative to surgical revision.

TORe was initially developed to induce weight loss by reducing the diameter of the GJA, thereby slowing gastric emptying and addressing the issue of a dilated, incompetent anastomosis [21]. Since rapid gastric emptying drives the symptoms of early and late dumping syndrome, TORe’s ability to enhance tissue competence and reduce GJA diameter, thereby delaying gastric pouch content release, accounts for its high clinical success rate and significant reduction in Sigstad scores [22]. In our meta-analysis, post-TORe Sigstad scores were significantly reduced compared to baseline, with a pooled mean 11.1-point reduction (*p* < 0.001). Most studies in this meta-analysis employed simple interrupted or figure-of-eight suturing patterns. While the purse-string technique has demonstrated superior weight loss outcomes compared to interrupted suturing [23], its impact on dumping syndrome efficacy remains unexplored. Future research comparing suturing techniques could clarify their relative effectiveness in this context.

Our updated meta-analysis builds upon the 2021 meta-analysis by Bazarbashi et al. [24], which had notable limitations, such as limited number of included studies and the incorporation of conference abstracts. In contrast, our study employed rigorous selection criteria, including only peer-reviewed studies to better evaluate bias risks and achieve more accurate effect estimates. We also incorporated newer studies not present in the prior analysis [13, 16, 20, 24]. Although Bazarbashi et al. assessed improvements in Sigstad scores, they analyzed this as a secondary outcome and included data from only two studies. In contrast, our meta-analysis evaluated Sigstad score improvement as the primary outcome, incorporating data from four studies.

Despite the encouraging results, several limitations should be acknowledged. First, the analysis is based exclusively on observational studies, with no randomized controlled trials (RCTs) available to date. This reliance on non-randomized data introduces potential risks of unmeasured confounding and selection bias. Consequently, high-quality prospective series and RCTs would confirm and strengthen the current evidence base. Second, although a random-effects model was employed to account for inter-study variability, significant statistical heterogeneity was observed across many outcomes. This may be attributable to differences in patient characteristics, baseline severity of dumping syndrome, definitions of clinical success, and variability in follow-up durations. Additionally, the definition of clinical success can be often subjective and inconsistent across studies, which may further confound the results. Third, the follow-up periods reported in the majority of the included studies were relatively short, limiting the ability to assess long-term durability and outcomes of TORe. Lastly, not all studies provided objective, quantitative pre- and post-procedure assessments using the Sigstad scoring system, which limits the ability to uniformly evaluate treatment response across cohorts.

Nevertheless, this study has important strengths. First, we included six studies with a total of 333 post-RYGB patients with dumping syndrome. Second, our meta-analysis evaluates the efficacy of TORe for dumping syndrome using objective criteria based on Sigstad’s scoring system. Lastly, our results remained consistent on sensitivity analysis for all relevant outcomes. By synthesizing available data from multiple international cohorts, this analysis provides valuable insight into the clinical utility of TORe in this challenging patient population.

In conclusion, our findings suggest that TORe is a safe and effective minimally invasive treatment for patients with medically refractory dumping syndrome. TORe should be considered before proceeding to surgical revision, which carries higher morbidity and technical complexity. Further prospective studies, particularly randomized trials with longer follow-up durations, are warranted to validate these findings.

## Supplementary Information

Below is the link to the electronic supplementary material.

ESM 1 Supplementary Material 1 (DOCX 58.2 KB)

## Data Availability

No datasets were generated or analysed during the current study.
