# Supplementary material for: Transoral Outlet Reduction for Dumping Syndrome After Roux-En-Y Gastric Bypass: a Comprehensive Systematic Review and Meta-Analysis
Source: Obes Surg. 2025 Sep 27;35(11):4620–7. doi: 10.1007/s11695-025-08275-9 (PMC12594707; doi:10.1007/s11695-025-08275-9)
Supplement: Supplementary file 1 — Supplementary Material 1 (DOCX 58.2 KB) [file 11695_2025_8275_MOESM1_ESM.docx]

# **Supplementary Appendix**

**Transoral Outlet Reduction for Dumping Syndrome after Roux-En-Y Gastric Bypass: A Comprehensive Systematic Review and Meta-Analysis**

**Supplementary Tables**

| Title | Page |
| --- | --- |
| Supplementary Table 1: Search strategy used in each database searched. | 2 |
| Supplementary Table 2: Quality assessment of the included studies in the meta-analysis. | 3 |

**Supplementary Figures:**

| Title | Page |
| --- | --- |
| Supplementary Figure 1: Leave-one-out sensitivity analysis for clinical success. | 4 |
| Supplementary Figure 2: Leave-one-out sensitivity analysis for repeat TORe. | 5 |
| Supplementary Figure 3: Funnel plot for publication bias assessment for clinical success. | 6 |

Supplementary Table 1: Search strategy used in each database searched.

| Database | Search Strategy | Articles retrieved |
| --- | --- | --- |
| PubMed | ((("transoral"[All Fields] OR "transorally"[All Fields]) AND ("outlet"[All Fields] OR "outlets"[All Fields]) AND ("reduction"[All Fields] OR "reductions"[All Fields])) OR ("gastrojejunal"[All Fields] AND ("revise"[All Fields] OR "revised"[All Fields] OR "revisers"[All Fields] OR "revises"[All Fields] OR "revising"[All Fields] OR "revision"[All Fields] OR "revisions"[All Fields]))) AND ("dumping syndrome"[MeSH Terms] OR ("dumping"[All Fields] AND "syndrome"[All Fields]) OR "dumping syndrome"[All Fields]) | 18 |
| Embase | ('transoral outlet reduction'/exp OR 'transoral outlet reduction' OR (transoral AND outlet AND ('reduction'/exp OR reduction))) AND ('dumping syndrome'/exp OR 'dumping syndrome' OR (('dumping'/exp OR dumping) AND ('syndrome'/exp OR syndrome))) | 57 |
| Web of Science | ((transoral outlet reduction) OR (gastrojejunal revision)) AND (dumping syndrome) | 26 |

Supplementary Table 2: Quality assessment of the included studies in the meta-analysis.

| Observational studies | Selection | | | | Comparability | Outcome | | | Overall risk of bias |
| --- | --- | --- | --- | --- | --- | --- | --- | --- | --- |
|  | Representativeness of the exposed cohort | Selection of non-exposed cohort | Ascertainment of exposure | Demonstration that outcome of interest was not present at start of study | Comparability of the cohorts on the basis of design or analysis | Assessment of outcome | Was follow up long enough for outcomes to occur | Adequacy of follow up cohorts |  |
| Lovis, 2024 | 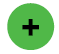 | 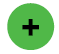 | 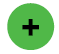 | 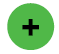 | 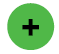 | 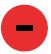 | 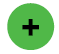 | 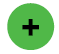 | 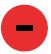 |
| Petchers, 2022 | 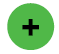 | 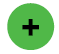 | 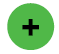 | 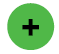 | 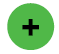 | 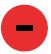 | 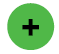 | 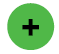 | 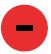 |
| Pontecorvi, 2023 | 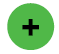 | 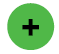 | 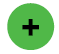 | 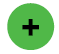 | 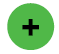 | 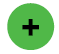 | 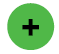 | 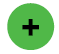 | 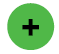 |
| Relly, 2021 | 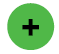 | 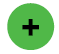 | 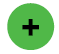 | 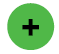 | 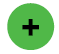 | 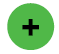 | 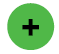 | 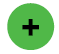 | 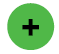 |
| Tsai, 2020 | 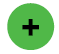 | 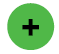 | 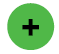 | 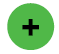 | 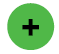 | 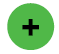 | 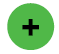 | 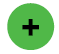 | 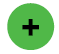 |
| Vargas, 2020 | 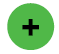 | 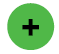 | 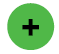 | 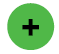 | 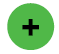 | 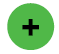 | 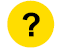 | 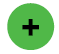 | 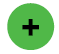 |

(
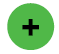
) low risk of bias, (
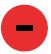
) High risk of bias, (
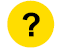
) Unclear risk of bias

Supplementary Figure 1: Leave-one-out sensitivity analysis for clinical success.


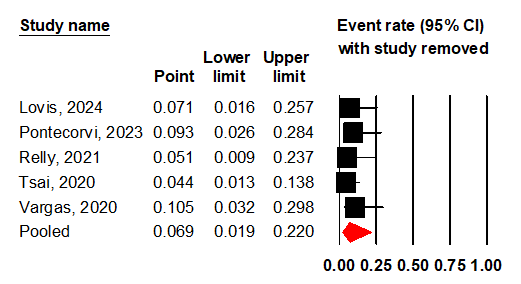


Supplementary Figure 2: Leave-one-out sensitivity analysis for repeat TORe.

Supplementary Figure 3: Funnel plot for publication bias assessment for clinical success.
